# Enhancing technology innovation performance through alliance capability: The role of standard alliance network and political skill of TMTs

**DOI:** 10.3389/fpsyg.2022.1008857

**Published:** 2022-10-03

**Authors:** Hong Jiang, Zhisong Wang, Sipeng Gao, Kaihua Chen, Fan Sheng

**Affiliations:** ^1^School of Business and Management, Jilin University, Changchun, Jilin, China; ^2^Department of Engineering, University of Cambridge, Cambridge, United Kingdom; ^3^Institute of Guangdong Hong Kong and Macao Development Studies, Sun Yat-Sen University, Guangzhou, Guangdong, China; ^4^Institutes of Innovation and Development, Chinese Academy of Sciences, Beijing, China; ^5^Department of International Development, Oxford University, Oxford, United Kingdom; ^6^School of Economics and Management, Harbin Engineering University, Harbin, Heilongjiang, China

**Keywords:** enterprise alliance capabilities, standard alliance networks, technology innovation performance, political skill, top management team

## Abstract

Given the increasing competition in standards, standard alliances have become a vital choice for enterprises to enhance their competitive advantage. In standard alliances, what decisions must top management teams make to help their enterprises improve their innovation performance? To answer this question, we draw on dynamic capability theory, social network theory, and high-level echelon theory to understand how alliance capabilities and standard alliance networks affect technology innovation performance. We collected questionnaire data from 465 manufacturing enterprises in China, and the empirical findings show that (1) enterprise alliance capabilities and standard alliance networks have a positive impact on technology innovation performance; (2) enterprise alliance capabilities and technology innovation performance are mediated by standard alliance networks; and (3) the political skills of top management teams strengthen this moderating model. The results of this study enrich the literature on standard alliances and provide a reference for enterprises in developing standard alliance strategies, cultivating alliance capabilities, and exercising the requisite political skills of top management teams.

## Introduction

Given the current global market turmoil and the accelerating updating of technology, it is difficult for most organizations to prosper if competing alone. An alliance is a contract-based organizational structure that breaks down the boundaries of an enterprise (Li, 2013) and helps enterprises get the technical knowledge and market resources required for development (Perez and Soete, 1988; Lee and Malerba, 2017; Zeng et al., 2019; Babu et al., 2020). For example, Lenovo, a core member of the Intelligent Grouping and Resource Sharing (IGRS) Alliance, and its alliance members jointly developed the “3C cooperative international standards,” which helped it capitalize on the 3C market, making many enterprises realize that significant economic benefits exist to cross organizational boundaries and in coordinating external innovation resources (Kumar et al., 2022). A standard alliance is a special type of strategic alliance between enterprises with independent research and development (R&D) capabilities and key technologies at their core, so that they and their partners can, together, initiate and spread standards (Axelrod et al., 1995). Standard alliances should not only focus on the effective combination of technology but also avoid opportunistic behavior. Therefore, standard alliances have important research and practical value. With the intensification of standard competition, the benefits of standard alliances have become the focus of business administrators (Lou et al., 2021; Wu and de Vries, 2021). For example, the IGRS alliance has 10 international standards. Its member manufacturers have more than 20 types of products based on the IGRS alliance standards, such as computers, cell phones, high definition network players, and wireless connectors, that are listed and sold, directly creating economic benefits of 2.28 billion yuan ($338 million).^1^ Although the role of standard alliances in promoting enterprise development is clear, how to help enterprises better compete through standard alliances remains unclear.

Some scholars have pointed out that the benefit of standard alliances depends on the differences in the enterprise alliance capabilities (ACs) (Anand and Khanna, 2000; Schilke and Goerzen, 2010). Existing literature on ACs and enterprise performance is abundant (Lavie and Rosenkopf, 2006; Schilke and Goerzen, 2010; Wassmer et al., 2017), and it is generally believed that there is a positive impact between the two. However, relatively few studies focus on technology innovation performance (TIP), and the division of AC dimensions depends slightly on one’s perspective: Schreiner et al. (2009) classified them as coordination, communication, and cohesion capabilities based on social relationships; and Simonin (1997) classified them as the capability to select alliance partners, negotiate the terms of cooperation, manage alliance operations, and terminate cooperation from a life cycle perspective. The present study considers that ACs are a type of dynamic capability. Therefore, we divide the AC dimensions from the perspective of dynamic capability and explore how each dimension impacts TIP. In addition, when exploring the relationship between ACs and TIP, scholars have analyzed them in the context of innovation capacity, resource integration, and organizational learning (see, e.g., Lambe et al., 2002; Draulans et al., 2003). According to dynamic capability theory, the effectiveness of ACs to improve performance needs to be complemented by organizational structure (Teece, 2007). However, several studies focus on the relationship between organizational structure in ACs and TIP, and among them, mainly revolve around networks of innovation alliances. Existing research on innovation alliance networks generally focuses on R&D alliances, patent alliances, and standard alliances (Shen and Shang, 2014; Wen et al., 2020; Fischer et al., 2021). The goal of R&D alliances is to promote the generation of new technologies and products; the goal of patent alliances is to patent new technologies; finally, standard alliances are a further step toward the marketization of technology standards, and technology standards are a higher outcome of R&D and patents (Hemphill, 2005). Thus, in contrast, the formation of standard alliance networks (SANs) requires more capacities from the enterprises themselves, but its social network function is stronger (Wen et al., 2020). Thus, this study explores the role of SANs in the relationship between ACs and TIP.

The top management team (TMT) is the core of enterprises (Hambrick, 2007), and a hot topic of current research is how enterprises can better use TMT capabilities to increase effectiveness. Some studies show that the external social relations and personal social networks of the TMT affect the enterprise strategy (Haunschild, 1993; McDonald and Westphal, 2003; Prashantham and Dhanaraj, 2010). Meanwhile, ACs require managerial acumen and coordination to integrate and plan (Ferris, 2005), and standard alliances are a risky enterprise strategy that requires excellent interpersonal skills from managers (Tocher et al., 2012). Wei et al. (2012) suggest that managers can facilitate risky and rewarding collaborations through political skills. Thus, membership selection, resource acquisition, and strategic change in the enterprise standard alliance strategy require that the TMT exercise its political skills. Research suggests that political skills are influenced by personality traits such as self-monitoring, initiative, and sense of responsibility (see, e.g., Epitropaki et al., 2016; Guo et al., 2020), whereas some studies explore how political skills affect leadership behavior, leader–member exchange, and relationship performance (see, e.g., Dahling and Whitaker, 2016; Ozturk and Emirza, 2021). Research on the moderating role of political skills has focused on examining the relationship between emotional performance, personality traits, strategy, and performance (Kimura, 2013; Liao et al., 2021), but research is lacking on the role of TMT political skills (TMTPSs) in standard alliances. Therefore, this study explores how TMTPSs impact the relationship between ACs and SANs.

Although the relationship between strategic alliances and enterprise performance has been thoroughly researched, it remains unclear if the relationship between alliance capabilities, alliance networks, and enterprise performance can be extended to the context of standard alliances. The present study uses 465 data points from manufacturing enterprises in seven cities in China, including Beijing, Shenzhen, and Shanghai, to explore the following empirical questions: First, in what ways can enterprises improve their alliance capabilities so that they can profit from standard alliances? Second, how can enterprises develop standard alliance networks and how can standard alliance networks help enterprises increase profits? Third, how can executives further improve performance by exploiting their own social networks and managerial capabilities? Finally, how can TMTs use their political skills to influence the creation of business-to-business partnerships?

## Theoretical analysis and research hypotheses

### Theoretical support

#### Theoretical framework

Dynamic capability theory suggests that dynamic capabilities help enterprises reconfigure and update existing resources as needed to respond quickly to external environmental changes and development opportunities (Teece, 2007), thereby gradually developing a core competitive advantage that distinguishes them from other enterprises. The present study proposes a research framework based on this theory. First, given that alliances are often considered as a way for enterprises to gain access to heterogeneous resources (Doz and Hamel, 1998; Das and Teng, 2000; Ko et al., 2020; Wen et al., 2020; Lin and Ho, 2021), ACs constitute a unique dynamic capability (Eisenhardt and Martin, 2000; Rothaermel and Deeds, 2006), which can improve enterprise innovation performance by changing environments (Hitt et al., 2001), reducing operating costs (Drnevich and Kriauciunas, 2011), and providing new decision-making options (Eisenhardt and Martin, 2000; Geleilate et al., 2021). Second, by adapting to changes in the external environment, enterprises can use this capability to make organizational changes to better align the enterprise with the dynamic market (Winter, 2003) and further promote enterprise development. Alliances are contract organizations form based on specific strategic goals such as resource sharing and risk sharing (Li, 2013), and enterprises can form their own alliance network structure according to their own interests to adapt to dynamic changes within the alliance and in the overall market. Third, enterprises constantly seek and use heterogeneous and complementary resources by optimizing partners and expanding the scale of their network, thereby maintaining a sustainable competitive advantage for their enterprise. In summary, based on dynamic capability theory, we argue herein that ACs are a key factor influencing TIP in standard alliances, and that the SANs are the critical factor in this relationship.

#### Concept definition

The core concepts of this study are defined as follows: First, ACs are defined as coordinated management capabilities that, to access heterogeneous resources, learn and change throughout the lifetime of alliances and alliance portfolios (Dubey et al., 2021), and identify the right time to reach an agreement with partners to achieve resource sharing and integration (Gulati et al., 2000). Second, according to the view of social network theory, inter-organizational alliances are network structures (Gulati, 1998; Peterman et al., 2020). In standard alliances, enterprises or alliances often aim to support a certain standard (or cooperation between different standards) as their common goal, and issues such as resource sharing, technology research, intellectual property rights, and benefit distribution in the process of standard development, implementation and diffusion when an agreement is reached, the set of formal partnerships established is the SAN. Third, technology innovation is defined as a series of activities related to technology innovation, such as organizing and coordinating the R&D, production, and marketing departments of an enterprise to produce technology ideas; implementing R&D; planning production; and performance evaluation (Damanpour, 1996). Finally, TMTPS refers to the capability of senior managers to obtain benefits by gaining the trust of others through effective interactions (Ferris, 2005), which is a type of social capital.

### Enterprise alliance capabilities and technology innovation performance

We view alliance capabilities as dynamic capabilities that highlight the significance of coordination, learning, and the reconfiguration of rules (Teece et al., 1997), which is a collection of multidimensional constructs based on organizational routines (Winter, 2003). Coordination routines are designed to allocate resources, assign tasks, and synchronize activities. Goerzen (2005) points out that not only is coordination within a single alliance necessary but also is vital for comprehensive governance of an enterprise’s entire alliance portfolio. Learning routines involve the process of generating new knowledge and new thinking, and inter-organizational learning can be effective in transferring knowledge between alliance partners (Dyer and Nobeoka, 2000). Some scholars view reconfiguration as a twofold element: sensing and transformation (Zahra et al., 2006; Teece, 2007). Sensing routines include scanning, searching, and exploring new opportunities, and transformation routines are designed to improve existing business logic to enable necessary adjustments. Therefore, the enterprise ACs are classified into five dimensions based on dynamic capability theory: inter-organizational coordination, alliance portfolio coordination, inter-organizational learning, alliance initiative, and alliance transformation (Schilke and Goerzen, 2010; Jiang et al., 2020). As a dynamic capability, the impact of ACs on the performance of enterprises participating in the process of standard alliances is explored from the following viewpoints:

First, inter-organizational coordination and alliance portfolio coordination can significantly reduce transaction costs. Schilke and Goerzen (2010) claim that it is unrealistic to establish a perfect-fit relationship between alliance partners. Strong coordination between enterprises facilitates the development of trusting relationships between partners and the signing of contracts that lead to the sharing of information between enterprises, reducing transaction costs and improving TIP (Hoffmann, 2007). The key point of alliance portfolio coordination is to guarantee the dependency between alliances, reduce conflicts of interest between organizations, and avoid repeating alliance activities (Goerzen, 2005), which further saves organizational resources for innovation activities.

Second, inter-organizational learning can help enterprises acquire, assimilate, integrate, and recreate knowledge and technology from other organizations. Zahra and George (2002) emphasize that enterprises could change traditional cognition by absorbing new knowledge, stimulating innovation consciousness, and improving innovation performance. Inter-organizational learning helps enterprises cultivate an atmosphere of innovation, consciously acquire and absorb what they lack in technology and knowledge, engage in more advanced fields, and collaborate with alliance partners to innovate and improve technology performance.

Finally, alliance initiative and alliance transformation can bring new and heterogeneous resources. Enterprises with initiative are better than followers at obtaining vital resources and gaining first-mover advantages (Sarkar et al., 2001). In addition, other scholars demonstrated that alliance initiatives had a positive and significant direct impact on enterprise performance (Mitrega et al., 2012; Leischnig and Geigenmueller, 2018; Inigo et al., 2020). The extent to which alliance initiative improves enterprise performance depends on the complementary capabilities of enterprises (Wei and Kem, 2019). Alliance transformation can help alliance partners constantly change an imperfect fit to achieve a better fit (Schilke and Goerzen, 2010), which in turn can be an effective way to improve TIP by obtaining new resources from quality partners.

In conclusion, in the alliances, alliance initiative can give enterprises first-mover advantages; inter-organizational coordination can maintain the long-term stability of alliance cooperation; alliance portfolio coordination can improve the quality of cooperation between alliances; inter-organizational learning can help enterprises acquire innovative resources; and alliance transformation can promote more consistent enterprise cooperation. We believe they all have a positive impact on TIP and are also important parts of ACs. Thus, we propose the following hypotheses:

*H1a–H1e*: Enterprise alliance capabilities (alliance initiative, inter-organizational coordination, alliance portfolio coordination, inter-organizational learning, and alliance transformation) have a positive relationship with technology innovation performance.

### Enterprise alliance capabilities and standard alliance networks

Venkatraman (1989) proposed that developing and deploying dynamic capabilities enables enterprises to continuously adapt their structure to the external environment. If the ACs of enterprises transform their social structure, enterprises would then have more priority opportunities for alliances, which, in turn, would help them expand their alliance networks (Rosenkopf et al., 2001). Andrevski et al. (2016) argue that standard alliances are typical alliances and are special strategic alliances of enterprises. Standard alliances are in fact an advanced form of alliance. The effect of enterprise ACs on SANs is revealed by the following two characteristics:

First, ACs can affect network structures. The process and results of inter-organizational learning increase the frequency of collaboration between enterprises in a network (Knight, 2002), thereby causing the organizational structure to evolve. In addition, the coordination capability of enterprises in an alliance strengthens the diversity and effectiveness of the alliance team (Zoogah and Peng, 2011); that is, enterprises can gradually expand the scale of the SANs through their capability to coordinate. Furthermore, alliance combinations involve a self-centered network focus (Kamal et al., 2021), and enterprises that coordinate alliance portfolios generally occupy the central position in the SANs (Ozcan and Eisenhardt, 2009).

Second, ACs can affect network relations. Enterprises that coordinate alliance portfolios maintain high-quality cooperative relationships between network members (Lechner et al., 2010; Kamal et al., 2021) and benefit from trusted partner network relations (Andrevski et al., 2016). At the same time, enterprises can constantly adjust their alliance strategies and search for suitable new opportunities, which allows them to continuously develop high-quality relationships in response to environmental changes (Ireland et al., 2003). Moreover, since establishing a standard has certain technology advantages related to timeliness, it is easier for the proactive enterprises in an alliance to gain the first-mover advantage by acquiring better partnerships and more resources (Sarkar et al., 2001).

In summary, in the alliance process, taking the initiative of forming an alliance has a positive effect on the timeliness of standard alliances. Inter-organizational coordination is conducive to the expansion of the alliance network, and the coordination of an alliance portfolio improves the quality of the network relationship. Inter-organizational learning also improves the enterprise’s position in an alliance network, and enterprises can also continuously improve the quality of cooperation networks through alliance transformation. Therefore, we believe that robust ACs have a positive impact on SANs. Thus, we propose the following hypotheses:

*H2a–H2e*: Enterprise alliance capabilities (alliance initiative, inter-organizational coordination, alliance portfolio coordination, inter-organizational learning, and alliance transformation) have a positive relationship with standard alliance networks.

### Standard alliance networks and technology innovation performance

According to social network theory, the social networks of team members exert a strong positive influence on innovation (Wang et al., 2020; Yang et al., 2022). Granovetter (1973) discusses the impact of social networks on innovation performance in terms of both structure and relationships. The former stems from the influence of organizational structure on enterprise performance. The establishment of an alliance network structure is favorable for the development of innovation activities (Ashraf et al., 2014). To create their own alliance network, enterprises are more likely, depending on their resources, knowledge, and innovation needs, to contract with other enterprises in the alliance (Fort, 2000). Network members are more closely connected, and resources such as information and knowledge circulate more easily in the alliance network, which helps enterprises obtain the required innovation resources (Inkpen and Tsang, 2005). Furthermore, a larger network scale of central enterprises is more conducive to the acquisition of resources through organizational learning and improves innovation performance (Rost, 2011; Tang et al., 2022). The latter is based on how organizational relationships affect enterprise performance. Burt (1992) proposed that, in a social network, some network members have direct and stable connections, while others have loose and inefficient connections. According to the “strong relationship” school of social network theory, the strength of network relationships fosters high trust and stable cooperation between network members, leading to an improved problem-solving capability. These characteristics facilitate the acquisition of resources in the network (Uzzi, 1997; Coleman, 1998) and improve enterprise innovation performance. The “weak ties” school believes that a network scale of weak ties is much larger than that of strong ties and that weak ties are more common among heterogeneous members. Therefore, better cross-border cooperation can be conducted through these weak ties (Granovetter, 1973; Friedkin, 1980). Although disagreements remain, most scholars agree that alliance networks promote innovation.

In addition, standards also affect technology innovation. Some scholars believe that, in the process of drafting documents and communicating and coordinating standards based on common technical requirements in standard alliances, enterprises inevitably obtain diversified information, knowledge, and other key resources, which positively affects innovation performance (Baum et al., 2000; Blind, 2001). However, other scholars believe that a virtuous cycle exists between technology standards and technology innovation only at the industry level; at the micro-level of enterprises, strong network externalities and path dependence related to standards increase conversion costs (Blind, 2001; Leiponen, 2008), thereby hindering enterprise technology innovation. Furthermore, Blind (2001) points out that technology standards have both beneficial and negative effects on TIP. The present study believes that standard alliances can help not only to decrease R&D expenditure and risk but also to open new markets and greatly alleviate the additional transformation cost of standard innovation. Therefore, there should be a positive relationship between the two. Thus, we propose the following hypothesis:

*H3*: Enterprise standard alliance networks have a positive relationship with technology innovation performance.

### The mediation effect of the standard alliance networks

Related studies generally confirm that enterprise ACs have a direct positive impact on innovation performance (e.g., Kale et al., 2002; Heimeriks and Duysters, 2007). Based on dynamic capability theory, the dynamic capability of an enterprise strengthens the organizational structure and thus achieves higher market efficiency (Rindova and Kotha, 2001). As a capability to expand external cooperation, ACs form a unique cooperation network through which enterprises can obtain the knowledge and resources needed for innovation and thereby improve innovation performance. Therefore, social networks form the critical path between ACs and innovation performance. Based on the characteristics of high goal consistency, knowledge aggregation, technology advancement, and management standardization of SANs, it is more conducive to enterprise technology innovation activities. This study considers the role of SANs as a mediator between ACs and TIP. Enterprise ACs promote the diversity of standards alliance partners, effective multilateral cooperation, dominance of cooperation, and quality of partnerships, resulting in a more efficient and creative SAN (Rosenkopf et al., 2001; Hu et al., 2020). Enterprises can reduce R&D costs and risks by collaborating through SANs and can avoid negative behaviors such as opportunism and freeloading (Hernandez et al., 2015).

Based on this analysis of enterprise ACs, SANs, and TIP, this study proposes that enterprises can use inter-organizational coordination and alliance portfolio coordination capabilities to access resources through SANs, use inter-organizational learning capabilities to absorb resources in SANs, and continuously respond positively to standard alliance opportunities and changes. Therefore, enterprises can continuously improve their TIP through SANs. Thus, we propose the following hypothesis:

*H4*: The enterprise standard alliance networks can serve to mediate between enterprise alliance capabilities and technology innovation performance.

### Moderating effect of the political skills of top management teams

Dynamic managerial capability theory argues that, without the social capital of individuals, enterprises will be unable to acquire, recombine, and release resources (Blyler and Coff, 2003). Political skills are characterized by social acuity, environmental adaptability, and interpersonal networks that constitute an important social capital for individuals. At the same time, high-level echelon theory suggests that individual leaders have limited knowledge and ability and usually need to make team decisions, so decisions are influenced by factors that characterize the TMT (Cho and Hambrick, 2006; Buyl et al., 2011). Therefore, we now discuss how TMTPS affects alliance strategy. The social acumen of the TMT affects how quickly an enterprise can respond to change; the environmental adaptability of the TMT can assist in coordination efforts; interpersonal characteristics influence how an enterprise perceives information and affect its flexibility to seize opportunities. TMTs with richer personal networks are more willing to embrace diversity so that enterprises with such teams more easily benefit from learning. Considering these factors, we argue that TMTPS can moderate the effectiveness of dynamic capabilities. Furthermore, as decision-makers in the enterprise, managers can rely on political skills to connect individuals in different networks within and across enterprises (Burt, 1992) and can influence the formation of alliance networks in the process of participating in the alliance.

Specifically, TMTPS can help the coordination capability between enterprises and alliances (Treadway et al., 2004; Ferris, 2005). Enterprises need strong alliance coordination skills to facilitate alliance agreements, and Lewicki et al. (2005) points out that TMTs with political skills are effective negotiators and therefore can facilitate alliance capabilities. The sensitivity and perception of the TMT also complement the ACs, which helps enterprises identify alliance partners and seize opportunities in a complex external environment (Ferris, 2005; Yang et al., 2021, 2022). A high social acumen of TMTPS translates into greater sincerity when dealing with relationships while simultaneously concealing intentions, which allows the enterprise to obtain resources through alliances while maintaining the relationship (Treadway et al., 2004). Thus, TMTPS can assist enterprises in obtaining critical resources (Ozdemir et al., 2016), allowing them to consistently improve their position in the network. At the same time, TMTPS includes the capability of senior managers to gain the trust of others through understanding (Ferris, 2005) and improve innovation cooperation, and thus has a positive impact on innovation behavior (Epitropaki et al., 2016). In conclusion, we believe that TMTPS positively influences enterprise ACs on the SANs. We therefore propose the following hypothesis:

*H5*: The political skills of TMT have a positive moderating effect between the enterprise alliance capabilities and the standard alliance networks.

As mentioned above, SANs are expected to moderate the impact of ACs on TIP because enterprises with powerful ACs will aggressively exercise their SANs, which enhances TIP. In many cases, enterprises with greater TMTPS are more likely to use resources from ACs to develop their alliance networks. It thus appears plausible to assume that increasing an enterprise’s participation in SANs through the interaction between ACs and TMTPS will increase its TIP. We therefore propose the following hypothesis:

*H6*: Standard alliance networks mediate the interactive effect of alliance capabilities and TMTPS on technology innovation performance.

**Figure 1:**
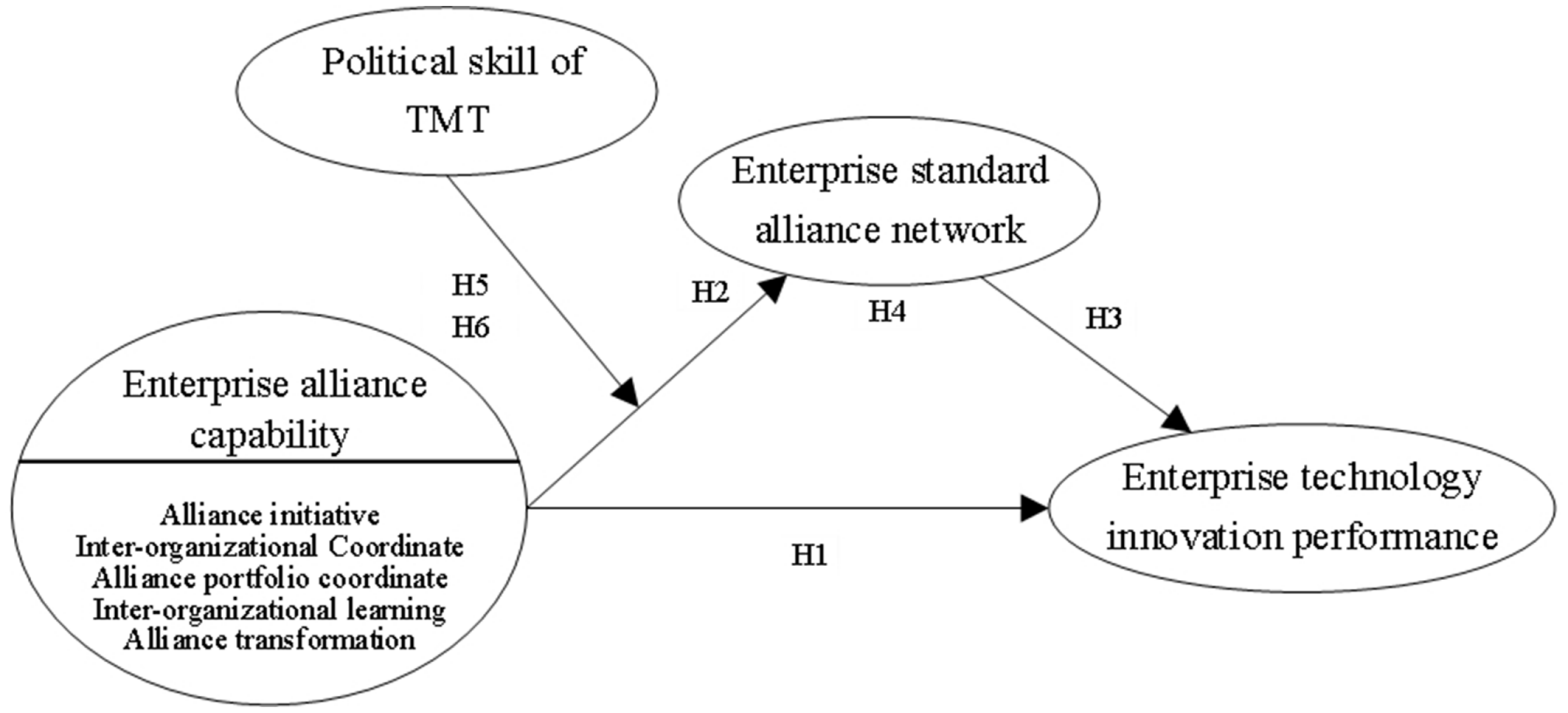
Conceptual model.

Based on hypotheses H1–H6, we propose the research model shown in Figure 1.

## Methodology

### Sample

In recent years, researchers have increasingly scrutinized Chinese standardization in the international market. The growth of Chinese standardization is characterized by a shift from relying entirely on international norms to autonomously generating its own standards, as well as a shift from government mandates to collaborations within industry. This development process involves numerous common and characteristic problems worthy of in-depth exploration. Therefore, data from the Chinese manufacturing industry have high research value.

This survey object is Chinese manufacturing enterprises that frequently engage in technology innovation activities. However, the survey is not limited to enterprises that have participated in standardization activities in collaboration with others. This factor serves as a control variable in this study, whereas only enterprise TMTs could fill out the questionnaire (i.e., chief executive officer, general manager, deputies, and others having decision-making and control power). Accounting for variances resulting from regional disparities in enterprise innovation, we leverage the “2019 China Science and Technology Cities Report,” which published the main selection of Chinese science and technology enterprises ranked No. 1 (Beijing), 2 (Shenzhen), 3 (Shanghai), 5 (Hangzhou), 20 (Chongqing), 29 (Changchun), and 30 (Harbin).

This research started in November 2019 and ended in July 2020 (8 months). It included several procedures such as pre-research, adjusting the questionnaire based on the pre-research results, finding appropriate respondents, releasing the online questionnaire, and collecting the questionnaire data over the last 2 months. The questionnaire was distributed mainly through online sample services, with a portion of it also available through social or government relations. The validity of the questionnaire is controlled by trap questions and time control, and the questionnaire is invalidated if information is missing or if there is an obvious regularity of options or contradiction of options, etc., ensuring high sample validity. This study focuses on the TMTs of manufacturing enterprises as the research object. Miller et al. (1998) and Janssen et al. (1999) argue that data from a single respondent can be representative of the team, and we include as respondents the CEO or other TMT members who may influence the strategic decisions of the enterprise, which is consistent with the approach adopted by much frontier research on TMT (Bedford et al., 2022; Xiaobao et al., 2022; Yu et al., 2022). The collected questionnaires were screened, 500 were sent out *via* the sample service, and 140 questionnaires were distributed through social networks. In the end, 640 questionnaires were distributed and 571 were returned (89.22% recovery rate), of which 465 were valid, giving an effective recovery rate of 72.66%. And we strictly control one enterprise to receive one copy of the data.

Samples were returned from Beijing (21.29%), Shenzhen (14.83%), Shanghai (12.90%), Hangzhou (15.91%), Chongqing (9.03%), Changchun (9.67%), Harbin (7.10%), and other cities (9.25%) such as Guangzhou, Wuhan, Changsha, Tianjin, Wuxi, and Suzhou. The TMTs are characterized by their age groups: 20–29 years old (4.95%), 30–39 years old (376.3%), 40–49 years old (23.87%), 50–59 years old (20.43%), and over 60 years old (13.12%). In terms of education level, the TMTs held bachelor’s degrees (44.95%), graduate degrees (50.75%), and other degrees (4.30%). In terms of employment experience, the TMTs have 0–4 years (3.01%), 5–9 years (10.11%), 10–14 years (23.44%), 15–19 years (27.10%), 20–25 years (19.78%), 25–30 years (12.04%), and over 30 years (4.52%). The basic sample was created by using SPSS26.0 software to sort the basic information from the 465 valid questionnaires gathered for this study. Table 1 shows the information distribution.

**Table 1:** Distribution table of sample characteristic information.

Control variables		Frequency	Percentage(%)
Position (POS)	Chief executive officer/Chairman/General manager	36	7.7
	Senior management of the R&D Department	160	34.4
	Senior management of the Sales Department	87	18.7
	Senior management of the Marketing Department	95	20.4
	Senior management in other departments	87	18.7
Scale (SCA)	Less than 100 people	77	16.6
	100–199 people	89	19.1
	200–499 people	148	31.8
	500–1,000 people	98	21.1
	More than 1,000 people	53	11.4
Industry (IND)	Electronic and communication equipment manufacturing	113	24.3
	Pharmaceutical manufacturing	115	24.7
	Medical equipment and instrumentation manufacturing	37	8.0
	Chemical manufacturing	49	10.5
	Computer, software, and office equipment manufacturing	70	15.1
	Aviation, spacecraft, and equipment manufacturing	11	2.4
	Automobile and transportation equipment manufacturing	24	5.2
	Other	46	9.9
Attribute (ATT)	State-owned enterprise	84	18.1
	Public institutions	13	2.8
	Joint venture	100	21.5
	Foreign company	28	6.0
	Private	234	50.3
	Other	6	1.3
Standard cooperation experience (STA)	Have	392	84.3
	No	73	15.7
	Total	465	100.0

Based on Harman’s single-factor test, the largest factor explained 30.037% of the variance, which rules out any common method bias. Before the regression analysis, we verified the multicollinearity of the independent variables. The VIF of all independent variables is less than the critical value of 10, so the independent variables are not multicollinear and regression analysis can be done.

### Measures

To design the measurement items in this study, we mainly used the mature scales developed and used by researchers internationally. Based on the research content of this article, we also revised some measurement indicators and then consulted experts in the fields of technology innovation management for the revised items. The questionnaire was improved based on expert feedback; the final questionnaire appears in Table 2. The questionnaire uses a Likert 7-point scale, where 1 is “very inconsistent” and 7 is “very consistent.”

**Table 2:** Reliability analysis of variables.

	Variables	Items	Cronbach’s α after deleting an item	Cronbach’s α
AC	AC1	AC11 The work of our company and alliance partners is coordinated with each other.	0.726	0.788
		AC12 We are sure that our company’s work is synchronized with our alliance partners.	0.740	
		AC13 There is a lot of communication and interaction between our company and alliance partners in most decisions.	0.668	
	AC2	AC21 Our company can coordinate with each other when participating in activities between different alliances.	0.809	0.839
		AC22 Our company can determine the synergy area of the alliance combination.	0.812	
		AC23 Our company maintains the interdependence between alliances.	0.780	
		AC24 Our company can determine whether there is overlap between different alliance partners.	0.780	
	AC3	AC31 Our company can learn from alliance partners.	0.742	0.811
		AC32 Our company has the management ability to absorb new knowledge from partners.	0.763	
		AC33 Our company has a complete inspection program to analyze the information obtained from alliance partners.	0.779	
		AC34 Our company has the ability to integrate existing knowledge and new knowledge.	0.766	
	AC4	AC41 Our company strives to seize the competitive advantage by intervening in the alliance.	0.772	0.801
		AC42 Our company often actively contacts those companies that have R&D plans or standardization plans.	0.740	
		AC43 Compared with competitors, our company is more forward looking and sensitive in the process of seeking alliance partnership.	0.746	
		AC44 Our company actively monitors the environment to determine the opportunities for alliances.	0.747	
	AC5	AC51 To improve the results of the alliance, our company is willing to put aside the terms of the alliance contract.	0.688	0.765
		AC52 When an unexpected situation occurs, our company is more willing to adjust the agreement with the alliance partner instead of insisting on the original provisions.	0.690	
		AC53 To respond to changing needs, flexibility is the characteristic of our company in the alliance management process.	0.676	
SAN		SAN1 Our company has many partners in the standard alliance network.	0.890	0.901
		SAN2 Our company’s standard alliance network is more diverse.	0.885	
		SAN3 Our company has a high reputation in the industry.	0.889	
		SAN4 Our company is often in the leading position in the standard alliance.	0.891	
		SAN5 Many alliance partners are willing to cooperate with our company in standardization.	0.884	
		SAN6 Our company and many partners in the alliance network have/are/plan standardization cooperation.	0.886	
		SAN7 Our company maintains frequent interactive communication with partners in the standard alliance network.	0.896	
		SAN8 There is a high degree of trust between our company and the standard alliance network partners.	0.894	
		SAN9 Our company maintains a long-term, stable, and win–win cooperation relationship with standard alliance network partners.	0.895	
TIP		TIP1 Our company has more advanced production equipment or technological processes.	0.880	0.897
		TIP2 The new products developed by our company have high technical content.	0.865	
		TIP3 Our company has a short average cycle for developing new products.	0.886	
		TIP4 The success rate of our company’s new product development is high.	0.884	
		TIP5 The new products developed by our company have a good market response.	0.876	
		TIP6 The new products developed by our company have a large market share.	0.880	
TMTPS		TMTPS1 Our company’s TMT is good at insight into the purpose and ideas of others.	0.853	0.874
		TMTPS2 Our company’s TMT is good at using words and actions to influence others and gain support.	0.853	
		TMTPS3 Our company’s TMT is sincere in words and deeds at work.	0.853	
		TMTPS4 Our company’s TMT can make most people feel comfortable and relaxed at work and is very good at winning everyone’s favor.	0.854	
		TMTPS5 Our company’s TMT spends a lot of time and energy establishing contacts with influential people.	0.851	
		TMTPS6 Our company’s TMT is good at using the network to make the work go smoothly.	0.850	

This study analyzes how enterprise ACs affect the mediation and dependent variables from the alliance. Based on the research of Schilke and Goerzen (2010), the ACs were divided into five dimensions: inter-organizational coordination (AC1), alliance portfolio coordination (AC2), inter-organizational learning (AC3), alliance initiative (AC4), and alliance transformation (AC5). The questionnaire was slightly adjusted and revised, and the final design measured 18 items. Drawing lessons from the SAN scale developed by Fang and Pigneur (2007), nine measurement items were designed, and the innovation performance scale developed by Prajogo and Ahmed (2006) was used to design six measurement items to measure TIP. The Leadership Political Skills Scale developed by Ferris (2005) and Zhang and Liang (2012) was used to design six measurement items to measure the TMTPS, such as the TMT’s use of networks and the work climate created by the TMT.

For the control variable, we relied on the research of Dai et al. (2017) and considered the characteristics of the industry in which the enterprise participated and the size and attributes of the enterprise. The experience of participating in standard alliances may affect the willingness to answer questions regarding the behavior of such standard alliances. Finally, based on the research of Hambrick and Mason (1984), the senior management positions were selected as the control variable.

To ensure the applicability of the scale for this study, we conducted a pre-investigation to test its reliability and validity. A total of 145 questionnaires were distributed in the pre-investigation, which produced 123 valid questionnaires (84.8%). The scale was then revised based on the results to form the final questionnaire for the formal study.

## Analyses and results

### Reliability and validity analysis

Reliability analysis uses Cronbach’s α coefficient to reflect the internal consistency of variables. Table 2 shows the results of SPSS processing. Cronbach’s α coefficients for all variables are greater than 0.7, indicating that the scales used in this study are reliable.

Next, we conducted a validity analysis. The measurement scale was subjected to a confirmatory factor analysis using AMOS 26.0, and the result conformed to the standard (*χ*^2^/DF = 1.774 < 3, CFI = 0.943 > 0.9, IFI = 0.943 > 0.9, TLI = 0.937 > 0.9, RMSEA = 0.041 < 0.08). Table 3 shows the aggregation validity test of each latent variable and the results. The standardized factor loading coefficient of each item on the corresponding latent variable is greater than 0.6, the composite reliability (CR) is greater than 0.7, and the average variance extracted (AVE) is greater than 0.5. Each fitting index satisfies the standard, the factor structure is verified, and the scale had good aggregation valid. Table 4 shows the results of the test for discriminant validity. The square root of each variable’s AVE exceeds the correlations, so the discriminant validity is high.

**Table 3:** Results of aggregation validity test.

Dimension	Items	Normalized factor loading	CR	AVE	Dimension	Items	Normalized factor loading	CR	AVE
AC1	AC11	0.730	0.791	0.558	SAN	SAN1	0.713	0.902	0.508
	AC12	0.713				SAN2	0.770		
	AC13	0.795				SAN3	0.731		
AC2	AC21	0.729	0.840	0.568		SAN4	0.705		
	AC22	0.722				SAN5	0.791		
	AC23	0.794				SAN6	0.770		
	AC24	0.767				SAN7	0.620		
AC3	AC31	0.758	0.812	0.520		SAN8	0.655		
	AC32	0.739				SAN9	0.640		
	AC33	0.685			TIP	TIP1	0.778	0.898	0.596
	AC34	0.699				TIP2	0.854		
AC4	AC41	0.651	0.802	0.504		TIP3	0.708		
	AC42	0.727				TIP4	0.728		
	AC43	0.729				TIP5	0.791		
	AC44	0.729				TIP6	0.763		
AC5	AC51	0.674	0.765	0.521	TMTPS	TMTPS1	0.748	0.875	0.538
	AC52	0.697				TMTPS2	0.745		
	AC53	0.790				TMTPS3	0.715		
						TMTPS4	0.729		
						TMTPS5	0.730		
						TMTPS6	0.732		

**Table 4:** Discriminant validity test table and correlation coefficient matrix.

	AC1	AC2	AC3	AC4	AC5	SAN	TIP	TMTPS
AC1	0.747							
AC2	0.574^**^	0.754						
AC3	0.582^**^	0.524^**^	0.721					
AC4	0.500^**^	0.519^**^	0.521^**^	0.710				
AC5	0.385^**^	0.437^**^	0.388^**^	0.452^**^	0.722			
SAN	0.580^**^	0.573^**^	0.571^**^	0.632^**^	0.493^**^	0.713		
TIP	0.528^**^	0.494^**^	0.571^**^	0.503^**^	0.381^**^	0.638^**^	0.772	
TMTPS	0.107^*^	0.154^**^	0.147^**^	0.192^**^	0.047	0.245^**^	0.194^**^	0.733

*p < 0.05, **p < 0.01, *N* = 465.

### Tests of hypotheses

To verify hypothesis 1, we constructed linear regression models 1 and 2. Table 5 shows the regression results, showing that the four dimensions of enterprise ACs (inter-organizational coordination, alliance portfolio coordination, inter-organizational learning, alliance initiative) are positively correlated with TIP (*β* = 0.173, *p* < 0.01; *β* = 0.126, *p* < 0.01; *β* = 0.291, *p* < 0.01; *β* = 0.166, *p* < 0.01 in Model 2). Therefore, H1a–H1d hold. The positive relationship between alliance transformation and TIP is weaker compared to the other four dimensions (*β* = 0.077, *p* < 0.1 in Model 2). Although it has not reached the 0.05 significance level, it is significant at the 0.1 critical significance level, so the positive relationship between alliance transformation and TIP is marginally significant. That is, during the alliance process, enterprises can promote TIP by improving the corresponding ACs, which verifies our previous conjecture.

**Table 5:** Results for hypotheses testing.

Variables	Dependent variable: TIP								Dependent variable: SAN									
	Model 1		Model 2		Model 3		Model 4		Model 5		Model 6		Model 7		Model 8		Model 9	
	β	*p*	β	*p*	β	*p*	β	*p*	β	*p*	β	*p*	β	*p*	β	*p*	β	*p*
POS	0.039	0.411	−0.005	0.884	0.018	0.618	−0.005	0.894	0.033	0.000	−0.002	0.952	−0.003	0.920	0.002	0.952	−0.001	0.979
SCA	−0.029	0.562	−0.092	0.019	−0.075	0.057	−0.092	0.012	0.071	0.493	0.003	0.941	−0.001	0.970	0.000	0.995	−0.007	0.836
IND	−0.068	0.156	−0.027	0.463	−0.025	0.505	−0.019	0.594	−0.068	0.16	−0.023	0.472	−0.028	0.389	−0.032	0.311	−0.028	0.357
ATT	0.053	0.281	−0.009	0.810	0.021	0.583	−0.008	0.815	0.051	0.155	−0.002	0.956	−0.007	0.838	−0.006	0.861	−0.005	0.867
STA	−0.093	0.062	−0.030	0.425	−0.031	0.422	−0.019	0.600	−0.097	0.304	−0.032	0.345	−0.030	0.372	−0.023	0.486	−0.030	0.346
AC1			0.173	0.000			0.107	0.024			0.186	0.000						
AC2			0.126	0.008			0.071	0.120			0.154	0.000						
AC3			0.291	0.000			0.233	0.000			0.162	0.000						
AC4			0.166	0.000			0.058	0.205			0.301	0.000						
AC5			0.077	0.066			0.022	0.577			0.152	0.000						
AC													0.738	0.000	0.717	0.000	0.675	0.000
SAN					0.639	0.000	0.358	0.000										
TMTPS															0.122	0.000	0.075	0.017
AC*TMTPS																	0.183	0.000
*F*	1.436		35.400**		53.828**		40.305**		2.208		57.762**		94.582**		85.726**		84.178**	
*R* ^2^	0.015		0.438		0.414		0.495		0.023		0.560		0.553		0.568		0.596	
Adjusted *R*^2^	0.005		0.426		0.406		0.482		0.013		0.550		0.548		0.561		0.589	

*p < 0.05, **p < 0.01, *N* = 465.

To verify hypothesis 2, we constructed multiple linear regression models (Models 5 and 6). Table 5 shows the regression results, that the five dimensions of enterprise ACs are positively correlated with SAN (*β* = 0.186, *p* < 0.01; *β* = 0.154, *p* < 0.01; *β* = 0.162, *p* < 0.01; *β* = 0.301, *p* < 0.01; *β* = 0.152, *p* < 0.05 in Model 6). Therefore, H2 is established.

To verify H3, we constructed Model 3. Table 5 shows the test results, which support H3, so the SAN is positively correlated with TIP (*β* = 0.639, *p* < 0.01). We have thus established H1–H3. To verify H4, we constructed Model 4 (*β* = 0.358, *p* < 0.01), which preliminarily verifies the mediation effect.

To confirm the mediating role of SAN between the ACs and TIP, we tested it with the SPSS bootstrap (*n* = 5,000). Table 6 shows the test results of the mediation effect (AC1 → SAN → TIP), which reveals direct effects (Boot 95% CI = [0.150, 0.346]), indirect effects (Boot 95% CI = [0.236, 0.372]), and total effects (Boot 95% CI = [0.453, 0.642]). This illustrates that SANs act as mediators between AC1 and TIP. Similarly, SANs act as mediators between the other four dimensions of ACs and TIP. Thus, this empirical research supports the hypothesis that the SANs mediate between enterprise ACs and TIP. This test thus establishes that enterprises can increase the success rate of SANs through ACs, and thereby establish long-term and stable cooperative relations and continuously diversify their alliance networks. Finally, through standard innovation cooperation activities, they can improve TIP.

**Table 6:** Bootstrap mediation effect test results.

	Hypothesis	Coefficient	Standard error	LLCI	ULCI
AC1 → SAN → TIP	Total effect	0.550	0.048	0.453	0.642
	Direct effect	0.249	0.051	0.150	0.346
	Indirect effect	0.301	0.035	0.236	0.372
AC2 → SAN → TIP	Total effect	0.493	0.045	0.406	0.538
	Direct effect	0.195	0.044	0.107	0.281
	Indirect effect	0.230	0.037	0.230	0.374
AC3 → SAN → TIP	Total effect	0.557	0.040	0.475	0.635
	Direct effect	0.302	0.043	0.220	0.387
	Indirect effect	0.255	0.035	0.190	0.327
AC4 → SAN → TIP	Total effect	0.475	0.046	0.384	0.566
	Direct effect	0.160	0.049	0.063	0.257
	Indirect effect	0.316	0.037	0.246	0.394
AC5 → SAN → TIP	Total effect	0.400	0.051	0.299	0.496
	Direct effect	0.100	0.044	0.013	0.186
	Indirect effect	0.299	0.037	0.227	0.372

**Figure 2:**
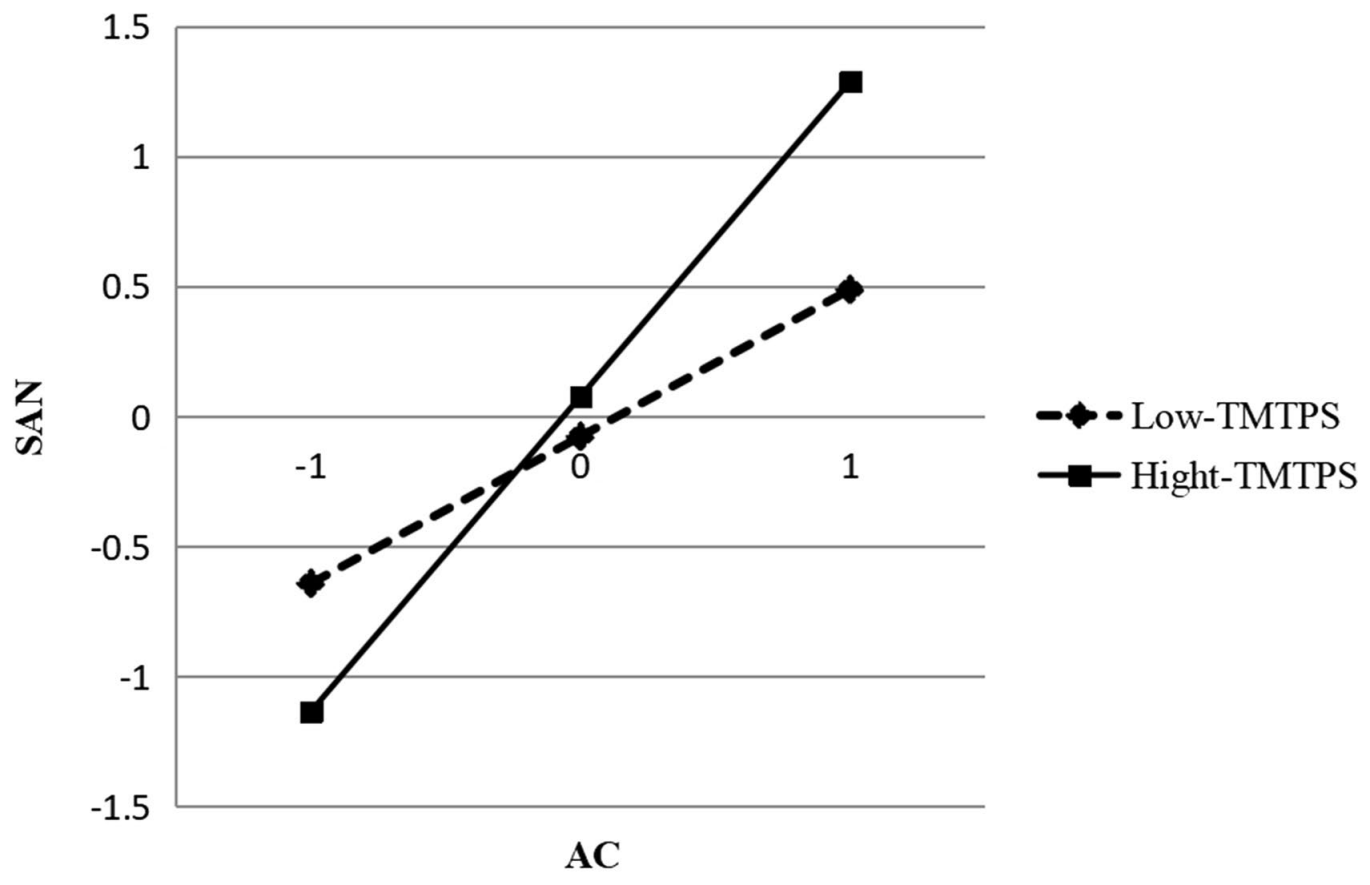
Moderating effect of TMTPS between AC and SAN.

To test H5, we constructed Models 7–9. The results of this empirical research are given in Table 5 and show that TMTPS plays a positive role in moderating the relationship between enterprise ACs and SANs (*β* = 0.183, *p* < 0.01 in Model 9). Thus, H5 is supported. Based on the results, we graph the moderating effects in Figure 2. When the TMT has strong political skills, it can promote the full use of enterprise ACs, connect more potential alliance partners, promote long-term, stable, and harmonious partnerships between enterprises and alliances, and maximize alliances through inter-organizational learning while calmly adapting to alliance changes, etc. This can further improve the SAN structure and relationships.

Table 7 shows the results of the moderated mediation test, as TMTPS in the low, middle, and high level, the indirect effect of SANs on enterprise TIP through ACs exists. The coefficients are 0.138, 0.279, and 0.470, respectively, and the confidence interval does not include zero, and the difference in indirect effects is significant. Thus, H6 is accepted.

**Table 7:** Moderated mediating effect test result.

	TMTPS	Effect	Boot SE	Boot LLCI	Boot ULCI
Indirect effect	Eff1: Low(–1SD)	0.138	0.053	0.043	0.256
	Eff2: Mid(0)	0.279	0.052	0.178	0.383
	Eff3: High(+1SD)	0.470	0.091	0.289	0.647
Pairwise contrasts between conditional indirect effects	Eff2-Eff1	0.141	0.039	0.060	0.215
	Eff3-Eff1	0.331	0.105	0.117	0.524
	Eff3-Eff2	0.190	0.066	0.056	0.314

## Discussion and conclusions

### Discussion of major findings

As noted above, the standard alliance strategy is important for innovation, and AC is a key factor in the success of standard alliances. Based on dynamic capability theory, high-level echelon theory, and social network theory, this research develops a theoretical framework to examine these ideas. The results of empirical tests lead to some important conclusions.

First, this study confirms that ACs are positively correlated with TIP in a significant way. Consistent with the findings of Luvison and de Man (2015), ACs positively affect innovation performance. The results reveal that enterprises that organize alliances or participate in alliances gain first-mover advantages in leading technology standards and seizing market shares for new products, thereby fundamentally promoting the rapid improvement of enterprise TIP (Sarkar et al., 2001). Furthermore, the promotion effect of ACs involves five dimensions: alliance initiative, inter-organizational coordination, alliance portfolio coordination, and inter-organizational learning, and all are positively correlated with TIP (note that alliance transformation has a marginally significant effect).

In addition, the research data support the positive correlation between ACs and SANs, and SANs and TIP. This result shows that, in standard alliances, enterprises can identify alliance opportunities by monitoring the external environment and internal needs of enterprises and actively partaking in alliance activities. The result of such a strategy is that their alliance network relationships will continue to be diversified. Furthermore, the gradual deepening of cooperation and the selection and integration of alliance members also promotes the continuous optimization of the SAN structure.

However, regarding the relationship between alliance networks and TIP, the results of the present study are inconsistent with those of Goerzen et al. (2007) and Duysters and Lokshin (2011), who find that alliance networks negatively affect innovation performance, but support the findings of Hoffmann (2007). The positive effect of SANs on enterprise innovation performance is reflected in the fact that a reasonable use of alliance networks is conducive to enterprise growth (Hoffmann, 2007), which plays a decisive role in enterprise innovation performance. At the same time, the confirmation of the mediation effect of SANs further demonstrates that the strategic choice of the enterprise to partake in standard alliances should be accompanied by the corresponding organizational structure change to better assist the enterprise to innovate in standard alliances.

Finally, this study confirms the moderating effect of TMTPS. Consistent with previous results, the present results indicate that politically skillful individuals perform better in interpersonal interactions (Ferris, 2005) and play a crucial role in the formulation of enterprise strategy. That is, when the TMT has strong political skills, it can promote the full use of enterprise ACs, connect to more alliance partners, promote long-term, produce maximum-value alliance interactions through inter-organizational learning, adapt to the modifications of the alliance, etc., all of which improves the SAN structure and relationship.

### Theoretical contributions

This study is built from three different research contributions: enterprise technology innovation, standard alliances, and dynamic managerial capabilities in alliances.

Our model identifies the important factors that promote innovation and provides a new perspective for exploring the relationship between standard alliances and innovation performance. As digitization progresses, more and more enterprises want to lower innovation risk through alliances, whereas the existing literature provides a relatively limited understanding of the factors that contribute to innovation success through participation in standard alliances. This study thus cultivates the traditional link between ACs and enterprise performance by finding SANs as a major mediator and TMTPS as a moderating element. As a result, this mediated-moderating model offers a better lens through which to study how ACs influence enterprise performance. In the process of exploiting ACs to promote innovation, the cultivation of corporate social relations and the characteristics of the TMT are crucial. In addition, this study is based on dynamic capability theory and social network theory, which allows us to better understand how building self-capacity and social network relationships contribute to innovation through the process of participating in alliances.

This study extends the research of dynamic capability theory and social network theory to the context of standard alliances, thereby enriching this literature. Due to the high knowledge and technology concentration and fierce competition and cooperation between members of standard alliances, the optimization of enterprise performance in standard alliances requires considering situations that are more complex. The present results show how enterprises can improve performance through participation in standard alliances. By using the theory of dynamic capabilities, the contribution of ACs to innovation performance was verified in a standard-alliance context. By using social network theory, the promotion of enterprise performance by SANs is verified.

Furthermore, this study integrates psychology and management to extend research on executive characteristics into the literature on standard alliances. This study thus enriches the research context of higher echelon theory by analyzing the relationship between TMTs and enterprise behavior in the context of standard alliances and confirms that TMTs ultimately impact organizational performance. Simultaneously, this study contributes to research on dynamic managerial capabilities by identifying important factors in the process of participating in alliances that determine enterprise performance and by exploring in-depth how dynamic managerial capabilities should be applied in enterprise standard alliance strategies. Finally, the data show that TMTPS, which is one of the dynamic managerial capabilities, positively moderates the relationship between ACs and SANs.

### Managerial implications

The results of this research provide insights into how enterprises can improve their technology innovation performance through participation in standard alliances.

First, to seek innovation and development, technology-based enterprises often choose strategic alliances, but the success rate of strategic alliances has not been high heretofore. Based on the above research results, we recommend that enterprises focus on cultivating full-cycle alliance capabilities and consider establishing internal departments responsible for strategic alliances. On the one hand, enterprises that cultivate full-cycle ACs and can undertake wall-chart construction for each alliance: grasp the market dynamics and the internal needs of the enterprise, master the financial and performance reporting (Kliestik et al., 2020), process alliance cooperation in real-time, and actively and promptly resolve emergencies. On the other hand, to continuously expand alliance networks, enterprises should not aim solely at the success of the alliance but instead develop more alliance opportunities through each partner while looking for long-term and stable partners in the process of innovation and cooperation.

Second, enterprises should deploy and actively participate in standard alliances as soon as possible; such alliances represent a mutually beneficial (win-win) cooperation model. Although the success rate of enterprise standard alliances is not high, the risks of alliances are slightly less than the risks of completely independent R&D, especially for small- and medium-sized enterprises. Large enterprises can also cooperate between alliances to integrate technology between different standards. During the COVID-19 epidemic, people’s lifestyles and consumption patterns changed drastically, forcing the business models of enterprises to adapt (Pop et al., 2022; Vinerean et al., 2022) and significantly increasing the business risks of enterprises. The present results indicate that the risks of standard alliances can be reasonably avoided. On the one hand, we analyze enterprise internal needs and assess the external environment to develop reasonable standard alliance strategies and avoid alliance risks. Before forming a standard alliance, based on their own resources and capabilities, enterprises should assess the needs of the standard alliance, according to the planning and goals of strategic development. In addition, the external environment should be analyzed through industry analysis, competitor evaluation, and social repercussions. Furthermore, enterprises should predict the process and outcomes of a standard alliance, identify possible risks, and provide a basis for the formulation of alliance strategies. On the other hand, enterprises should select partners with long-term-cooperation experience and common cooperation goals to form a standard alliance network for high-quality cooperation with minimal cooperation risks. In the process of daily cooperation, enterprises should follow long-term plans. They should choose alliance partners who can cooperate tacitly and who can be trusted, which minimizes the risk of opportunistic behavior in the operation of the alliance.

Third, when enterprises participate in standard alliances, TMTs should focus on their political skills. To create possibilities for TMTs to expand their related abilities, enterprises may need to invest more in related training. This could improve the quality of their political abilities and, as a result, improve the efficiency with which they employ alliance resources. Based on the findings of this study, we believe that, in standard alliances, the TMT should use consistent words and deeds and treat others sincerely to influence their partners while gaining their understanding and support and facilitating the smooth development of the alliance. In the negotiation of alliance agreements, TMTs should focus on their partner(s), gain insight into their ideas, empathize, establish a rapport, and strive to occupy a dominant position in communication. TMTs should also expand their network of relationships through alliances to facilitate information acquisition, innovation, and cooperation. By bringing political skills to bear, the TMT can play a greater strategic role in external innovation cooperation, internal management, and enterprise culture. In addition, in the context of the COVID-19 epidemic, the rise of mobile business platforms requires TMTs to digitally transform their political skills to continuously promote the healthy development of the alliance.

### Limitations and future research

Future research can remedy the following limitations of this study: First, we only tested our model on Chinese manufacturing enterprises. Since China is a market that has a distinctive institutional development, the extension of the results of this study to other countries may produce different results. Future research can test the model by taking samples from different markets, such as developing and developed countries. Second, this study mainly focuses on the two important variables of enterprise ACs and SANs, while ignoring other factors that can affect enterprise performance. Future research should explore the key factors in standard alliances, for example, network externalities (Au and Kauffman, 2001), absorptive capacity (Golgeci and Kuivalainen, 2020), competition and cooperation within alliance networks, knowledge searching (Yayavaram et al., 2018), characteristics of combined alliances (Bi et al., 2020), and other external factors; enterprise innovation ability and atmosphere, enterprise intelligence and social capital, enterprise internal collaboration (Wilden et al., 2013), human resource practices, and other internal factors. Finally, this research uses a questionnaire survey. Future studies should account for factors such as the willingness and behavior of the team and employees through cross-level analyses with an in-depth analysis of the operating and governance mechanisms of the standard alliances based on theories drawn from sociology and psychology.

## Data availability statement

The raw data supporting the conclusions of this article will be made available by the authors, without undue reservation.

## Ethics statement

Ethical review and approval was not required for the study on human participants in accordance with the local legislation and institutional requirements. Written informed consent for participation was not required for this study in accordance with the national legislation and the institutional requirements.

## Author contributions

HJ contributed to conceptualization, methodology, validation, supervision, project administration, and funding acquisition. ZW contributed to validation, formal analysis, writing-original draft, writing-review and editing and visualization. SG contributed to conceptualization, methodology, validation, formal analysis, investigation, writing-original draft, writing-review and editing, supervision, project administration, and visualization. KC contributed to writing-review and editing and project administration. FS contributed to validation and investigation. All authors contributed to the article and approved the submitted version.

## Funding

This research was supported by the National Natural Science Foundation of China (71774067).

## Conflict of interest

The authors declare that the research was conducted in the absence of any commercial or financial relationships that could be construed as a potential conflict of interest.

## Publisher’s note

All claims expressed in this article are solely those of the authors and do not necessarily represent those of their affiliated organizations, or those of the publisher, the editors and the reviewers. Any product that may be evaluated in this article, or claim that may be made by its manufacturer, is not guaranteed or endorsed by the publisher.

## Footnotes

^1^ http://www.igrs.org/
